# Contributions to Medicine, Surgery, and Ophthalmology

**Published:** 1846-01

**Authors:** 


### Art. XV.

*Beiträge zur Medizin, Chirurgie und Ophthalmologie.* Von Chr. Conr. Wuth, Dr. med., chirurgiæ et artis obstetriciæ, praktischem Arzte etc. in Hannover. Mit Abbildungen. *Berlin,* 1844.

*Contributions to Medicine, Surgery, and Ophthalmology.* By C. C. Wuth, m.d. With two lithographic Plates. *Berlin,* 1844. 8vo, pp. 134.

The first forty-five pages of this work are occupied with an essay, entitled "General reflections on medicine," the chief drift of which is to show the necessity of being well acquainted with the healthy actions of the body, before attempting the study of diseases. The rest of the work contains a collection of interesting cases, partly medical, partly surgical, which have occurred to the author during a practice of many years' duration.

Although we cannot compliment Dr. Wuth on his style of writing, which is both rugged and prolix, the valuable facts contained in his work will well repay the pains of perusal, as may partly be judged from the following case, the only one on record, as far as we know, of a polypus being extirpated from the frontal sinus, and which we shall present to our readers in an abridged form. Its treatment does great credit to Dr. Wuth's skill:

Case.—"A boy, aged 10 years, was put under Dr. Wuth's care by his parents, with the remark, that for the disease of the eye, under which the child had already laboured nine years, they had sought the aid of many practitioners, who had used for it both internal and external remedies without relief. Their means were now exhausted, and the disease seemed to be incurable. During all this time the boy had suffered from severe pain in the head, and had enjoyed little or no rest, night or day. The left eye was so entirely pushed forwards out of the orbit, that it lay on a level with the back of the nose. Its lateral displacement projected it so much over the cheek-bone that, viewed in front, it hid the neighbouring side of the face. The displacement downwards brought the eye into a line with the point of the nose. For the last three years the eye had closed less and less completely, and the lids now covered it so imperfectly that the cornea, with a circumference of sclerotica four lines broad, remained constantly exposed,—whence a perpetual flow of tears. The strong orbicularis palpebrarum, whose annular fibres surrounding in concentric lines the protruded eye, seemed to press it still more out of the orbit. The extraordinary development of the muscle seemed to arise from its antagonistic action against the protruding swelling. A large, deep ulcer of the cornea threatened a speedy bursting, and total disorganization of the eyeball. A convolution of varicose veins of the conjunctiva covered the visible part of the sclerotica.

"The regions of the frontal and nasal bones were greatly protruded; while the eyeball had gradually quitted its natural place, in proportion as the orbit had become contracted, by the pressure exercised upon its constituent bones. The left side of the nose formed a flat surface along with the back of the nose, and a firm obstacle presented itself to the finger passed into the left nostril. From the stretching of the skin, the left eyebrow was separated widely from the right, and dragged downwards. The skin itself was thickened, and doughy to the touch, while at the outer-under part of the eyebrow was a small opening, from which, on pressing the surrounding region, a whitish mucous fluid walled out.

"As to the exciting cause, all that could be said was that nine years before, the child had a red-spotted eruption, with cough and severe headache (perhaps measles,) and that the present disease had for several years been increasing, under almost constant pain in the head and many sleepless nights. Although the boy was much emaciated, and had a livid countenance, he seemed to possess a good original constitution.

"Dr. Wuth's diagnosis led him to a favorable prognosis. He showed the patient to the Hanover Medical Society. The views of the members on the nature of the disease were much divided. Some thought it medullary fungus, other exostosis, osteo-sarcoma, a tumour in the orbit, &c. Dr. Wuth gave it as his opinion that a large polypus occupied the frontal sinus, which he trusted he should be able to remove by an operation. To this he proceeded next day, by making first a vertical incision, two inches long, from the root of the nose upwards through the soft parts, and then a horizontal one, also two inches long, close above the eyebrow. He next dissected off the triangular flap thus formed, so far as to permit the frontal sinus to be trepanned. In the middle of the superciliary arch was a small hole in the bone, scarce a line in diameter, opening into the sinus, and explaining the source of the fluid already mentioned, which had kept the soft parts in an inflamed and fungous state. This state of the soft parts caused a considerable bleeding in the course of the two incisions. In consequence of the great dilatation of the sinus, it was necessary to make two openings into it with a small trephine, whereupon an immense quantity of polypi protruded, being connected together like grapes, and covered with a milk-white fluid. Dr. Wuth removed the greater part of the polypi with cutting instruments. After washing away the slimy, milk-white fluid, he remarked that the polypi internally were of a yellow colour and partly transparent, and that within each polypus there ran two or three vessels, giving off branches on all sides. The upper portion of the cavity contained mucous polypi, which felt partly soft, and yielded readily to pressure, but were partly firmer and of cellular texture, so that out of the cells a white, slimy fluid could be pressed. In the middle of the cavity the morbid product became denser and more opaque; posteriorly and towards the parietes of the cavity, it assumed a fibrous structure. The cavity terminated towards its inner and outer sides in small sinuses, or cellular spaces, in which the polypi lay firmly imbedded.

"Dr. Wuth next directed his attention to radically destroying the remainder of the disease, attached to the parietes of the cavity and contained in its cellular spaces, selecting such means as were not likely to act injuriously on the very thin osseous partition, separating the cavity from the brain, and which was felt to yield to gentle pressure with the finger. He felt with his finger, lightly applied, the pulsation of the brain, and was afraid lest so thin an osseous partition might yield to the movements of the brain, now that the counter-pressure was removed by emptying the cavity of its contents. He now bored through the osseous parts from the cavity of the nose to the frontal sinus, as the ethmoid cells, conchæ, &c., and introduced a silver canula, so that the fluid collected in the sinus might flow off unconfined. Further, to remove the stillicidium lacrymarum which had arisen from the compressed state of the nasal duct, he perforated the nasal bone from without inwards, so as to meet (if we understand him right,) the artificial canal just mentioned, and here put in a second tube. After a time, being convinced that the tears had a free passage, he removed the tube from the artificially-formed nasal duct; and feeling assured of the healthy state of the frontal sinus, he removed also the canula, leaving the opening into the sinus to close.

"The healing of the parts occupied twelve months, the frontal sinus being by that time considerably lessened in all directions, and the eye having partially returned into the orbit. The ulcer of the cornea cicatrized soon after the operation, leaving a leucoma, which during the succeeding years diminished considerably, so as to permit the sight to improve. The physiognomy of the body became much more tolerable. From the first night after the operation he enjoyed sleep, such as he had not had for years. The first six weeks he spent chiefly in sleeping and eating, whereby his exhausted constitution was speedily restored. After this Dr. Wuth saw the lad twice a year, and each time found the disfigurement to be diminished.

"With regard to the applications made to the parietes of the sinus, in order to restrain any new growth, Dr. Wuth was afraid to use lunar caustic or kali causticum, and employed laudanum, Goulard's extract, and kreosote, pencilling the parietes with a mixture of equal parts of the two former, and then applying a salve, composed of an ounce of zinc ointment and ten drops of kreosote, spread on lint."

From the above case, Dr. Wuth draws the following physiological and pathological conclusions :

1. The possibility of the continuance of the power of vision under gradual traction and elongation of the optic nerve.
2. The enormous enlargement of which the frontal sinus is capable,—equal in this case to the containing of three hen's eggs.
3. The return of this osseous cavity almost to its normal form.
4. The complete division of the supraorbital and frontal nerves did not in this case injure vision.
5. Pain is not a pathognomonic sign of the malignant or benignant nature of a swelling, a fact illustrated also by cases of induration of the mammæ and other organs, which by their spontaneous disappearance show their non-malignant nature.

---

